# Automated Mineralogy Analysis of the Apollo 17 73002 Continuous Core Thin Sections Using QEMSCAN Mapping Techniques

**DOI:** 10.1029/2024JE008359

**Published:** 2024-11-30

**Authors:** S. K. Bell, K. H. Joy, M. Nottingham, R. Tartèse, R. H. Jones, J. J. Kent, C. K. Shearer, the ANGSA science team

**Affiliations:** ^1^ Stratum Reservoir AS Sandnes Norway; ^2^ Department of Earth and Environmental Sciences University of Manchester Manchester UK; ^3^ School of Geographical & Earth Sciences University of Glasgow Glasgow UK; ^4^ GeoControl Systems Inc. Jacobs JETS Contract NASA/JSC Houston TX USA; ^5^ Department of Earth and Planetary Science Institute of Meteoritics University of New Mexico Albuquerque NM USA; ^6^ Lunar and Planetary Institute Houston TX USA

**Keywords:** Apollo 17, ANGSA, QEMSCAN, core, regolith

## Abstract

The Apollo 17 73001/73002 double drive tube, collected at the base of the South Massif in the Taurus‐Littrow Valley, was opened in 2019 as part of the Apollo Next Generation Sample Analysis program (ANGSA). A series of continuous thin sections were prepared capturing the full length of the upper portion of the double drive tube (73002). The aim of this study was to use Quantitative Evaluation of Minerals by SCANing electron microscopy (QEMSCAN), to search for clasts of non‐lunar meteoritic origin and to analyze the mineralogy and textures within the core. By highlighting mineral groups associated with meteoritic origins, we identified 232 clasts of interest. The elemental composition of 33 clasts was analyzed using electron microprobe analysis that revealed that all clasts were of lunar origin, suggesting that any meteoritic component in the regolith material we studied is not present in the form of lithic clasts. In the process of searching for meteorite fragments, we also identified a number of clast types including a group with highly magnesian olivine compositions (Fo_92.2‐96.5_). We extracted raw pixel data to investigate changes in mineralogy with depth, used QEMSCAN processors to separate and group individual clasts based on mineralogy, and determined variations in particle size with depth. Our results show a decreasing abundance of glass and agglutinate clasts with depth, associated with a higher soil maturity in the upper portion of the core. The lack of stratigraphy and dominance of non‐mare clasts is consistent with the landslide origin of the material from the South Massif.

## Key Points

- Automated mineralogy can be used for the rapid identification of minerals of interest in Apollo 17 core samples
- Over 200 clasts of potential meteoric origin were identified; of these, 33 clasts were subject to further elemental analysis
- We collected a wealth of textural and mineralogical data from across the full length of the 73002 Apollo 17 continuous core thin sections

## 1 Introduction

**Figure 1:**
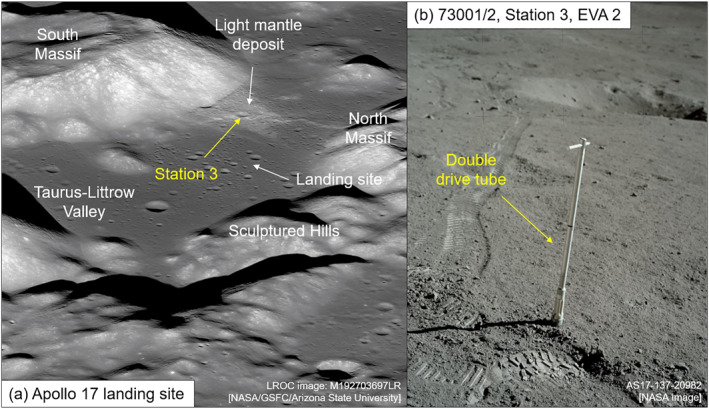
(a) Lunar reconnaissance orbiter (LROC) narrow angle camera (NAC) oblique view of the Apollo 17 landing site in the Taurus‐Littrow Valley. Apollo 17 Station 3 (indicated with yellow arrow) is found within the region covered by the light mantle deposit. (b) Photograph taken by astronauts on the lunar surface showing the collection of the double drive tube sample 73001/2 at Station 3.

The Apollo 17 landing site is located on the south‐eastern edge of Mare Serenitatis in the area known as the Taurus‐Littrow valley (Figure 1a). At station 3 (Figure 1b), during the second extra vehicular activity (EVA 2) of the mission, a 4 cm diameter double drive tube (73001/73002) was used to collect a sample of the top ∼70 cm of lunar regolith (Wolfe et al., 1981). The area from which the sample was taken is known as the light mantle deposit, located at the base of the South Massif (Jolliff et al., 2020; Wolfe et al., 1981). The light mantle deposit is interpreted to be a landslide material that collapsed off the face of the South Massif (Lucchitta, 1977; Magnarini et al., 2021, 2023; Schmitt et al., 2017). The trigger event that caused the landslide is currently unknown.

The lower portion of the double drive‐tube, 73001, was sealed under vacuum on the lunar surface (Allton, 1989; Butler, 1973). The upper portion of the double‐drive tube, 73002, was returned un‐sealed (Allton, 1989; Butler, 1973). For nearly 50 years, the core samples remained unopened and stored by NASA in pristine conditions, in anticipation of future technological advances. Our study is part of the Apollo Next Generation Sample Analysis (ANGSA) initiative to analyze the continuous thin sections from the 73002 drive tube opened by NASA in 2019 (Petro, 2020; Shearer et al., 2022; Simon et al., 2020). Some key goals to be addressed by the ANGSA initiative include (but are not limited to): understanding the stratigraphy within the core samples, identifying the lunar lithologies present within the regolith, and identifying interesting clasts within the samples for further analysis to address outstanding lunar science questions around lunar avalanche deposits and the evolution of the lunar regolith through time (Shearer et al., 2022).

Our primary scientific interest in the ANGSA core samples was to identify mineral and lithic fragments that could potentially have been delivered to the lunar surface as meteorites. The lunar surface has been bombarded by impacts for billions of years and as such one possible lithology present within the Apollo 17 73002 core sample could be remnants of impactor material. When an impactor hits the Moon, the most likely outcome is that the impactor is vaporized by the energy released; however, fragments may survive if the impactor was traveling at a low velocity of <4 km/s (Armstrong, 2010; Crawford et al., 2008) or impacted at an oblique angle of <10° (Bland et al., 2008; Joy et al., 2016; Pierazzo & Melosh, 2000; Schultz & Crawford, 2016; Svetsov & Shuvalov, 2015). The composition and type of material could potentially provide insights into the causes of bombardment through time (e.g., Ryder, 1990; Wetherill, 1981) and small body migration and transfer of meteoritic material through the Solar System (e.g., Joy et al., 2016, 2020; Strom et al., 2005).

Exogenously‐derived components preserved in the lunar regolith may include fragments of meteorites and/or micrometeorites that have survived the impact process. Such material would potentially contain minerals largely absent in lunar samples (e.g., hydrated minerals, uncommon sulphides, and Na‐rich phases) or minerals with non‐lunar chemical compositions. We know from previous analysis of Apollo samples and lunar meteorites that it is possible to preserve fragments of ordinary chondrites (Day et al., 2006; Joy et al., 2012; Liu et al., 2009; Warren & Wasson, 1979), carbonaceous chondrites (Fitzgerald & Jones, 1977; Joy et al., 2013, 2020; McSween, 1976; Wood et al., 1971; Zolensky, 1997; Zolensky et al., 1996), enstatite chondrites (Haggerty, 1972; Rubin, 1997), iron meteorites (Goldstein et al., 1970; Jolliff et al., 1993; McKay et al., 1971; Quaide & Bunch, 1970), mesosiderites (Wood et al., 1971), and achondrites (Joy et al., 2014).

Previous attempts to identify meteoritic material in lunar samples have involved time intensive manual searching of samples using a range of microscopy, imaging, and elemental analysis techniques (Day et al., 2006; Fagan et al., 2015, 2016; Haggerty, 1972; Joy et al., 2011, 2012, 2014; Warren & Wasson, 1979; Wood et al., 1971). Quantitative Evaluation of Minerals by SCANing electron microscopy (QEMSCAN) is a non‐destructive automated mineralogy system that uses energy dispersive X‐ray spectrometry (EDS) to produce mineral phase maps of samples. Processors within the QEMSCAN software can be used to extract further information from the mineral phase maps, including highlighting phases of interest (e.g., Bell et al., 2020). As such, QEMSCAN provides a method of extracting a large amount of mineralogical information from the Apollo 17 continuous core sections and provides rapid identification of potential extra‐lunar meteoritic material. Here we present the first QEMSCAN analysis of the Apollo 17 73002 continuous core thin sections. We use the QEMSCAN data to help identify potential clasts of meteoritical origin and further investigate clasts of interest further using electron probe microanalysis (EPMA).

We also use QEMSCAN mineral phase maps and pixel data to determine how the lunar mineralogy and clast types vary with depth within the core. As the 73002 core sampled the light mantle deposit, an area of landslide material from the South Massif, the absence or presence of any stratigraphy would be significant. The landslide material also provides a unique opportunity to investigate highland material shed from the South Massif mixed within the basaltic regolith from the valley floor (e.g., Schmitt et al., 2017) and to investigate the proportions of each lithology present (e.g., Simon et al., 2024). In addition, clasts such as agglutinates, which form by melting of the lunar soil during micrometeorite impacts, provide a measure of soil maturity and space weathering. Here we present variations in mineralogy, clast type abundance and particle size with depth within the 73002 core.

## 2 Samples and Methods

### 2.1 Samples

Thin sections were prepared at the NASA Johnson Space Center Apollo Curatorial labs (Gross et al., 2023). The 4 cm diameter 73002 core is comprised of loose regolith material, which was initially manually dissected in several vertical horizons (“passes”) (Gross et al., 2023). After dissection, the remaining 25% of the core was impregnated with epoxy and then sawn in half down the cores long axis to produce two sets of 30 μm thick, thin sections (Gross et al., 2023). Each set of four continuous thin sections spans a length of approximately 18.4 cm, encapsulating the full length of the 73002 core. In this study, we analyzed thin sections 73002,6011 to 73002,6014. The length values for each of the thin sections start at the top of the 73002 core with 73002,6011 (0–4.7 cm) and follow on sequentially through thin sections 73002,6012 (4.8–9.5 cm), 73002,6013 (9.6–14.2 cm), to the end of 73002,6014 (14.3–18.4 cm). Compression of the core occurred during collection and extrusion (Gross et al., 2023). In addition, part of the material in the bottom part of the 73002 tube was lost during collection on the lunar surface (Gross et al., 2023). An interval of approximately ∼1 mm was lost between each consecutive thin section due to sawing of the sample during the thin section making process (Gross et al., 2023). As such, the depth measurements for thin sections in this manuscript refer to the depth from the top (0 cm) of the extruded 73002 core material to the bottom of the material (18.4 cm), and do not refer to sampling depth within the lunar regolith. In relative terms, 73002,6011 represents the shallowest sampled material, and 73002,6014 the deepest material.

All 73002 continuous core thin sections were imaged using plane‐polarized light (PPL), cross‐polarized light (XPL), and reflected light using a Keyence VHX‐7000 digital microscope at the NASA Johnson Space Center Apollo Curatorial labs. Full resolution images are available in an accompanying data repository (https://doi.org/10.48420/c.7090330). Thin sections were then carbon coated at the University of Manchester prior to analysis using electron microscopy methods.

### 2.2 QEMSCAN

QEMSCAN analysis was conducted using an FEI QUANTA 650 field emission gun (FEG) scanning electron microscope (SEM) at the University of Manchester that is equipped with a single Bruker XFlash energy dispersive X‐ray spectrometer (EDS). An accelerating voltage of 25 kV and a 10 nA beam current were used to map the samples in field image mode with a step‐size (i.e., pixel size) of 5 μm between individual data collection. Qualitative major element chemical distribution maps can also be extracted from the QEMSCAN data set. All of the sample element maps are available in full resolution in an accompanying data repository (https://doi.org/10.48420/c.7090330).

During analysis, the FEI QEMSCAN software compares the EDS spectra and backscattered electron (BSE) image brightness collected at each individual measurement point on the sample to a customizable database of mineral definitions known as the Species Identification Protocol (SIP) list (e.g., Gottlieb et al., 2000; Pirrie & Rollinson, 2011). The BSE brightness was calibrated prior to analysis in the QEMSCAN software using quartz, copper, and gold standards, and the EDS spectra were acquired using 1,000 counts per pixel analyzed. Each SIP file within the list includes properties specific to a certain mineral, including elemental ranges, X‐ray count thresholds, and BSE brightness. The relative heights of the collected EDS spectra are compared to standard peak height information stored within the SIP definition, with element proportions reported out of 125% to account for low counting statistics. A mineral is assigned to each pixel on a first match basis when the collected data (EDS AND BSE brightness values) fall within the bounds of a mineral definition in the SIP list.

For this study, we used an updated version of the SIP list used by Bell et al. (2020) that was specifically established for lunar samples, referred to as the “Lunar SIP list.” The creation of the Lunar SIP list involved the adjustment or removal of standard mineral definitions in the master database provided by FEI, such as hydrous or ore minerals, to better reflect minerals commonly found in lunar samples. Changes to the original Lunar SIP list (Bell et al., 2020) include the addition of several minerals commonly found in asteroid‐sourced meteoritic material, and the refining and splitting of the glass chemical range definition to allow for determination between different glass types found in lunar samples (i.e., volcanic, or feldspathic or mafic impact derived glasses). These details can be found in Supporting Information S1. Every effort was made to include as many relevant mineral definitions as possible, but the Lunar SIP list is by no means exhaustive. Compositional ranges between two end member phases for certain minerals are accounted for within the Lunar SIP list. For example, olivine compositions from fayalite to forsterite end members are binned into one of nine synthetically generated definitions based on ideal mineral compositions, encompassing a continuous range from Fo_10_ to Fo_90_ (named sequentially as “Olivine Fo10” to “Olivine Fo90”). The Lunar SIP list also incorporates mixed phases, to account for when a measurement happens to be taken from a grain boundary that can produce a mixed EDS signal of the two adjacent phases. Mineral identification may also be affected by the degree of terrestrial alteration of a sample and sample preparation quality, although for the 73002 samples in this study it was not an issue, as the samples are pristine lunar material prepared into highly polished thin sections.

Mineral phase maps were initially classified in the iExplorer QEMSCAN software using the primary Lunar SIP list. Secondary SIP lists allow for the grouping of mineral definitions into a smaller number of broad mineral categories, for example, augite, pigeonite, and enstatite, under the phase name “pyroxene.” Additional secondary SIP lists were created that showed broad mineral groups (e.g., pyroxene, olivine, feldspar, etc.) and to pinpoint specific minerals of interest, such as those potentially associated with asteroidal meteoritic origin (e.g., Fe‐metal, metal‐sulphides, Mg‐rich pyroxene (>En_80_) and olivine (>Fo_80_), and Na‐rich feldspars). Phase maps showing the secondary SIP list groupings are available in an accompanying data repository (https://doi.org/10.48420/c.7090330).

The QEMSCAN software allows filters, processors, categorizers, and formulas to be applied to the data to determine additional properties of the sample. Here, we used the “area % of sample” calculation (total number of pixels of a mineral phase as a percentage of total number of pixels within sample) to provide an estimate of the modal mineralogy of each of the thin sections. Pixel data can also be extracted, which provides raw data for each of the pixels within a mineral map including raw elemental data, mineral classification, and location within the sample (*x* and *y* co‐ordinates). This enabled the calculation of modal mineralogy variations with depth at set intervals (i.e., 1 mm) across the full length of the 73002 core.

Additional processing steps were required to extract data for individual clasts from the QEMSCAN mineral maps. The Particulator processor in the QEMSCAN software can be used to identify and separate non‐touching clasts and the Touching Particles processor can be used to “separate” particles that are touching so that each particle can be parameterized for phase type and shape, for example, However, as the samples are incredibly complex materials with many touching clasts of various shapes and sizes, the use of the Particulator and Touching Particle processors alone was insufficient to separate individual clasts.

Another way to help distinguish between clasts is to consider variations in the EDS spectral count rate across the analyzed area. The count rate at the edge of a clast or in a crack is generally lower than that at the rest of the clast. As such, areas of low count rate can be used to highlight the boundaries of clasts. Areas of low count rate (pixels with a count rate of <20,000) were filtered out from the original phase maps, which helped to remove areas between clasts (cracks, small matrix particles). This resulted in fewer clumps of interconnected clasts being identified by the Particulator processor and in turn made touching clasts more easily identifiable using the Touching Particles processor (see Supporting Information S1). The Touching Particles processor was able to separate most clasts; however, some clasts remained connected. No additional manual separation was undertaken, to not introduce any human bias (i.e., it is easier to accurately identify larger grains that are touching compared to smaller touching grains). Another limitation of this clast separation method is that a small amount of material may be lost from the clast perimeter. Therefore, we only conducted further processing (e.g., clast classification) on clasts with an equivalent circle area of more than 150 μm, as the modal mineralogy and size of small particles are more affected by this method of processing than the larger particles. The Injector processor was also used to inject areas of internal background within a clast with a dummy mineral definition to prevent the Touching Particles processor from incorrectly splitting vesicular or partially fractured clasts into multiple smaller clasts.

Once the clasts were separated, the particle size was measured using an equivalent area circle calculation. The output of this step was used to determine grain size distributions for each thin section (both including and excluding clasts <150 μm for comparison). For clasts >150 μm, a categorizer was used to split the clasts into clast type groups based on modal mineralogy/occurring phases only. As such, the clast type groups vary from those used in other studies of 73002 (Cato et al., 2022; Simon et al., 2022, 2024), where clasts were manually identified to allow texture to be taken into account. The clast type groups and definitions are provided in full in Supporting Information S1. Shape, size, and *x*‐*y* co‐ordinates for each of the individual clasts within a sample were exported, allowing clast type distribution with depth to be calculated.

### 2.3 Scanning Electron Microscopy

Additional high resolution BSE maps of the continuous core sections were also collected using the FEI QUANTA 650 FEG SEM at the University of Manchester. An accelerating voltage of 15 kV and a dwell time of 10 µs were used to produce maps at a resolution of ∼1 μm per pixel. The BSE maps were collected in a single session to maintain a consistent contrast and brightness between the BSE maps of each thin section. Full resolution BSE maps of each sample can be found in an accompanying data repository (https://doi.org/10.48420/c.7090330). The high spatial resolution BSE maps were used along with the QEMSCAN phase and elemental maps to identify clasts of interest for further investigation via electron probe microanalysis.

### 2.4 Electron Probe Microanalysis

A Cameca SX100 instrument was used to measure the major element compositions of silicates, metals, sulphides, and glasses at the University of Manchester. Points and line profiles were collected across minerals within clasts of interest to help classification and identification of their sources. Analyses were carried out with a 15 keV accelerating voltage, 20 nA beam current, and a spot size of 1 μm for mineral and metal analysis and a defocussed 20 μm spot for glass analysis. The elements analyzed in the silicate minerals and glasses were Na, Al, Mg, Si, K, Ca, Ti, Cr, Mn, Fe, Ni, and Co. The elements analyzed in metals and sulphides were Fe, S, Ni, Co, Si, Zn, Cu, Mg, Ti, P, and Cr. Data were considered good if the totals fell within the region of 98%–102%. The EPMA data for each clast of interest are reported in full in the accompanying data repository (https://doi.org/10.48420/c.7090330) along with details of the standards used, detection limits, and associated measurement errors.

## 3 Results

**Figure 2:**
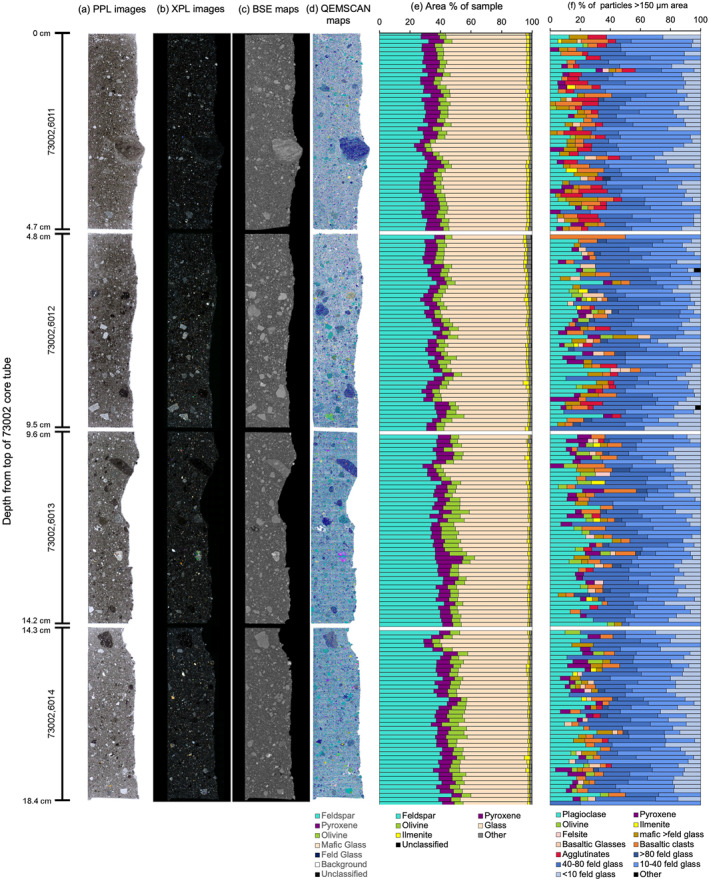
(a) Plane‐polarized light (PPL) and (b) cross‐polarized light images of the 73002 continuous core samples. (c) High resolution BSE images and (d) QEMSCAN mineral phase maps (full mineral list) for each thin section. A key showing broad colors for the main mineral groups in the QEMSCAN mineral phase map is given. (e) A plot showing the variation in mineral proportions (area % of sample) with depth for each of the four thin sections. Pixel data was binned into 10 mm intervals down the length of each core. For clarity, only broad mineral groups from the secondary mineral list (feldspar, olivine, ilmenite, pyroxene, glass, other, and unclassified) are shown. (f) A plot showing the variation in clast type (for particles >150 μm) with depth for each of the four thin sections using 1 mm bin intervals. Thin sections are ordered by depth from the top of the 73002 core tube (0–18.4 cm), as shown by the scale down the left‐hand‐side, which is the same for all images and plots. For individual full resolution images, please see the accompanying data repository (https://doi.org/10.48420/c.7090330).

Imaging and numerical data, collected for each of the four 73002 continuous core thin sections, are summarized in Figure 2. The thin section images and mineral maps are displayed in depth order from the top of the 73002 core tube (section 73002,6011) to the bottom (section 73002,6014) (Figure 2). Raw elemental data from each pixel (pixel data) were exported and binned into 10 mm depth intervals to show the fine scale variations in mineralogy across the thin sections. The identified phase proportions correlate well with what is visually observed in the images of the thin sections. For example, thin section 73002,6011 shows a large (∼8 mm in size) impact melt breccia clast (right‐hand side of thin section approximately 3 cm down), which correlates with an increase in glass abundance measured over the same interval in the QEMSCAN phase map (Figure 2). The data showing variations in clast type (for particles >150 μm) with depth (Figure 2) also shows the same correlation with observable clast type variation in the thin section images and mineral maps.

### 3.1 Mineralogy

In Figure 2e, the modal mineralogy is shown per 10 mm interval across the four thin sections. In Table 1, the mineralogy data are summarized and shown as a single value per mineral within the secondary mineral list for the full area of each individual thin section. This highlights broader mineralogical trends for each thin section, showing how the mineral proportions vary with depth on a larger (per thin section) scale.

**Table 1:** Normalized Modal Mineralogy per Sample Expressed as “Area % of Sample”

Sample	Plagioclase	Pyroxene	Olivine	Glass	Ilmenite	Other	Unclassified	Total
73002,6011	28.88	8.09	4.94	54.74	1.61	1.52	0.21	100
73002,6012	32.73	7.03	5.59	50.86	1.45	2.02	0.32	100
73002,6013	37.43	8.24	5.40	46.19	1.20	1.39	0.15	100
73002,6014	38.74	8.21	5.84	44.38	1.22	1.44	0.17	100

*Note.* The values given are for each thin section as a whole. Abundances are given for the broader mineral groups in the secondary mineral list. Abundances for the full primary list are given in the accompanying data repository (https://doi.org/10.48420/c.7090330).

The largest variations in the proportion of minerals across the four thin sections are seen in the abundance of plagioclase and glasses. The proportion of plagioclase increases with depth from ∼29% in 73002,6011 to ∼39% in 73002,6014. The proportion of glass decreases with depth from ∼55% in 73002,6011 down to ∼44% in 73002,6014 (Table 1). The highest proportion of ilmenite (1.61%) is observed in thin section 73002,6011, the shallowest of the four thin sections (Table 1). The proportion of ilmenite decreases with depth, to 1.45% in 73002,6012 and then to 1.20% in 73002,6013. The proportion of ilmenite in 73002,6014 (1.22%) is like that of 73002,6013 (1.20%). Olivine proportions per thin section vary from 4.94% to 5.84%, showing a trend of increasing proportion with depth (albeit the total variation in abundance is <1%). Pyroxene proportions remained consistent across the four samples (7%–8%).

The proportion of “Other” minerals varies from 1% to 2% in each sample. This “Other” mineral group includes accessory phases such as metals, sulphides, phosphates, zircon, and spinels. Each of the four samples contained a minor amount (<0.4%) of unclassified material. These pixels likely represent cracks and fractures within the samples and other areas of low count rate.

### 3.2 Clast Type Variation With Depth

Individual clasts were identified and separated from the QEMSCAN mineral maps using a combination of processors within the QEMSCAN software. In order for the categorizer function in the QEMSCAN software to work, the definitions for each clast type group had to be a combination of numerically definable inputs. As such, we were limited to using modal mineralogy as the only factor robust enough to reliably distinguish between different groups of clasts. This is in contrast to manual categorization studies (e.g., Cato et al., 2022; Simon et al., 2022, 2024), which take into account the texture within a clast to identify different lunar lithologies. As a result, the clast type groups reported here do not necessarily directly correlate with literature definitions of different lunar rock types. The clast type groups and definitions we used are provided in full in Supporting Information S1. For reasons outlined in Section 2.2, the automated separation and categorization of clast types was only conducted on clasts >150 μm. We note that automated clast separation and classification was conducted after the search for meteorite fragments was concluded; therefore, the >150 μm threshold did not influence the search for meteorite fragments.

**Figure 3:**
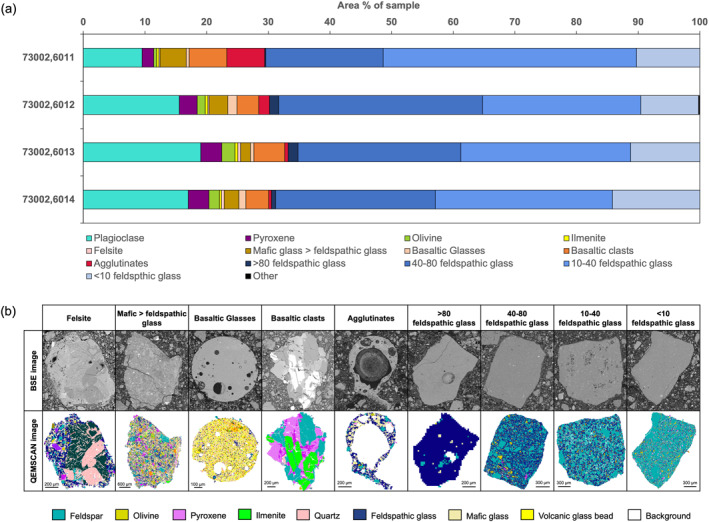
(a) Bar charts showing variability in clast type abundances (clasts >150 μm) for the four continuous core thin sections. Clast types include monomineralic clasts (i.e., plagioclase, pyroxene, olivine, and ilmenite), felsites, agglutinates, basaltic clasts, basaltic glasses, and a number of categories classifying the breccia clasts based on the proportion of mafic and feldspathic glass present. (b) QEMSCAN and corresponding BSE images of example clasts from each of the categories. The images are scaled to illustrate the characteristics of each group; as such, the scale bar varies between categories. A simplified QEMSCAN legend is provided to highlight some of the key mineral phases. A full description of the classification criteria for each category is provided in Supporting Information S1.

Monomineralic clast types include fragments of plagioclase, pyroxene, olivine, and ilmenite. The proportion of plagioclase mineral fragments increases with depth in the sample from ∼9.5% in 73002,6011 to ∼17% in 73002,6014, peaking at ∼19% in 73002,6013 (Figure 3). The proportion of pyroxene (∼2%–3.5%) and olivine (∼0.5–2%) mineral fragments varies to a smaller degree than plagioclase fragment abundance. The only variation of note is that the lowest pyroxene and olivine abundances are found in 73002,6011 (Figure 3). All four samples contain ∼0.5% ilmenite mineral fragments.

The proportion of basaltic clasts varies between thin sections from ∼3.5% to 6% and the proportion of basaltic glass fragments (including basaltic spherules) varies from ∼0.5%–1% (Figure 3). Thin section 73002,6011 contains the highest proportion of basaltic clasts at 6%, whereas at deeper depths in the core, the proportion of basaltic clasts is <5%.

All thin sections show a population of breccia clasts. These breccias are formed of a mixture of glass and mineral phases, in which the ratio of mafic glass to feldspathic glass varies (Figure 3). For clasts in which feldspathic glass % > mafic glass %, we identified four different groups containing different proportions of feldspathic glass compared with mineral/lithic fragments: >80% feldspathic glass, 40%–80% feldspathic glass, 10%–40% feldspathic glass, and <10% feldspathic glass (Figure 3). Collectively these four groups comprise up to 67%–70.5% of each thin section. The smallest proportion of these feldspathic breccia clasts is those with >80% feldspathic glass (<2%), followed by clasts with <10% feldspathic glass (∼9%–14%). The two dominant breccia clast types with feldspathic glass are those with 10%–40% feldspathic glass (∼26%–41%) and 40%–80% feldspathic glass (∼19%–33%). We also identified a population of breccia clasts which contained a more mafic glass than feldspathic glass. The abundance of clasts with mafic glass % >feldspathic glass % is highest (∼4%) in sample 73002,6011, and lowest (∼1.5%) in 73002,6013 (Figure 3).

The proportion of agglutinates continuously decreased with depth in the thin sections from ∼6% in 73002,6011 to ∼0.5% in 73002,6014 (Figure 3). Felsite clasts are present in all four thin sections, although they comprise a relatively minor proportion (<0.5%) of the total thin section area. A small proportion of clasts (<0.2%) does not fall under any of the clast classifications (“Others”).

### 3.3 Particle Size Variation With Depth

**Figure 4:**
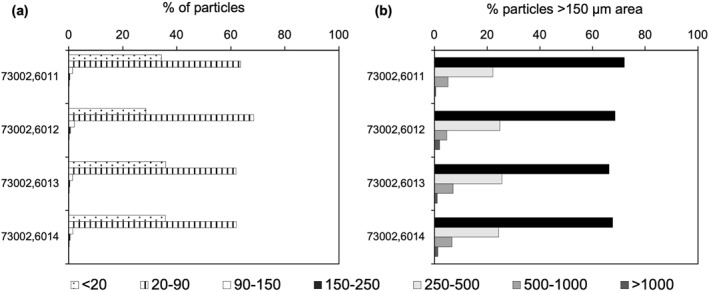
(a) Bar chart showing clast size distribution as percentage of total particles within each size bin per thin section. All four samples are dominated by clasts <20 μm and 20–90 μm in area. All size bins are represented in (a), but size bins of more than 150 μm make up too small of a percentage to be seen on this plot. Tabulated data can be found in the accompanying data repository (https://doi.org/10.48420/c.7090330).

Individual clasts were binned into size fractions of <20, 20–90, 90–150, 150–250, 250–500, 500–1,000 and >1,000 μm based on those previously used in other Apollo 17 73002 studies (Cato et al., 2022; Simon et al., 2022, 2024). All thin sections are dominated by 20–90 μm clasts (>60% of particles), followed by <20 μm clasts (Figure 4). This result is expected based on the fine‐grained nature of the regolith material surrounding the larger clasts (Figure 4), which visually makes up a larger proportion of the sample.

When looking in detail at the proportion of clasts >150 μm in area, all samples show the same trend of decreasing abundance with increasing grain size fraction (Table 2). The percentage of particles within each of the size bins remains consistent between samples with 66%–72% of particles 150–250 μm in area, 22%–26% of particles 250–500 μm in area, 5%–7% of particles 500–1,000 μm in area, and 1%–2% of particles >1,000 μm in area (Table 2).

**Table 2:** Overview of the Total Number of Particles per Sample and % of Particles per Sample Within Each of the Size Fractions

	Sample	Particle area (μm)							Total	Total >150
		<20	20–90	90–150	150–250	250–500	500–1,000	>1,000		
No. of particles	73002,6011	58,886	109,107	2,451	756	233	54	5	171,492	1,108
	73002,6012	28,282	67,705	2,007	636	230	44	18	98,922	928
	73002,6013	46,449	80,089	1,871	584	226	62	9	129,290	877
	73002,6014	45,882	79,457	1,900	601	216	59	12	128,127	907
% of particles	73002,6011	39.40	58.45	1.51	0.46	0.15	0.04	0.00	100	0.65
	73002,6012	34.82	62.26	1.98	0.65	0.22	0.05	0.02	100	0.94
	73002,6013	40.66	57.17	1.49	0.44	0.17	0.05	0.01	100	0.68
	73002,6014	40.41	57.37	1.51	0.48	0.17	0.05	0.01	100	0.71

### 3.4 Clasts and Mineral Phases of Potential Meteoritic Origin

A secondary mineral list was used to highlight minerals within the QEMSCAN mineral phase maps that are potentially associated with an asteroidal or planetary (i.e., non‐lunar) meteoritic origin. This list includes phases such as Fe‐metal, metal‐sulphides, Mg‐rich pyroxene (>En_80_) and olivine (>Fo_80_), and Na‐rich feldspars. We acknowledge that this approach might introduce bias in that it does not contain the total budget of known meteorite or planetary minerals, but notably covers some of the main mineral types found in a wide range of meteorite groups (Rubin & Ma, 2017). Full resolution QEMSCAN phase maps with the meteoritic origin secondary mineral list are available in the accompanying data repository (https://doi.org/10.48420/c.7090330).

Several hundred occurrences of minerals of interest were identified in clasts across the four thin sections. After identification, a manual review of each clast was then carried out to exclude endogenous occurrences of highlighted minerals of interest (e.g., an Fe‐sulphide grain in a mare basalt clast, or a Mg‐rich olivine in a lunar troctolite). This approach involved the cross‐examination of other mineral phase maps and element maps (e.g., Mg, Fe, Al, Ca, etc.) within the QEMSCAN data set for each thin section.

**Figure 5:**
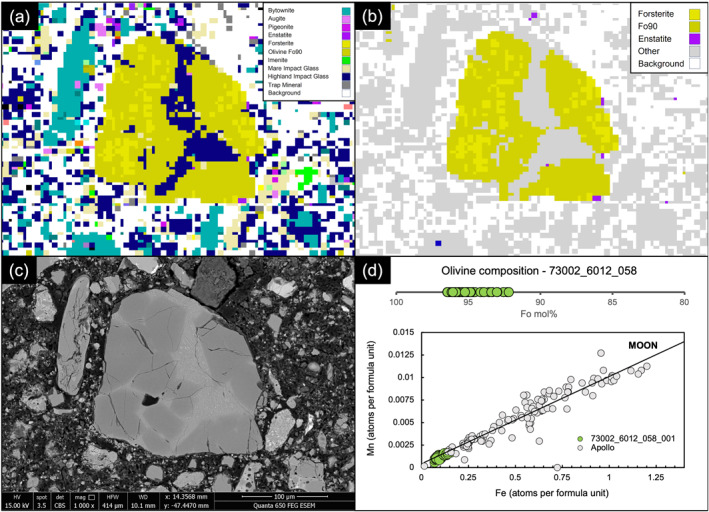
Depiction of the steps used to highlight clasts of interest using QEMSCAN and the further analysis steps taken to identify the origin of the clasts, using clast 73002_6012_58 as an example (where “_058” refers to our own internal clast numbering system). (a) Full Lunar SIP list QEMSCAN mineral phase map of clast 73002_6012_058. (b) The secondary mineral list containing minerals of potential meteoritic origin highlighted 73002_6012_58 as having forsteritic olivine. Please note that QEMSCAN mineral phase names such as “Olivine Fo90” represent a compositional bin (see Section 2.2) and do not represent an EPMA determined mol% Fo_#_. (c) A high‐resolution BSE image of the clast, used to further inspect texture and to determine if further investigation using EPMA was warranted. (d) Olivine composition EPMA data for clast 73002_6012_058, showing highly forsteritic values of up to Fo_96_ that have Mn versus Fe ratios consistent with a lunar origin. Clast 73002_6012_58 is thought to be a type of Mg‐suite material. Apollo samples shown in gray, are Apollo minerals in different lunar rock types from the database of Jolliff et al. (2006).

A total of 232 clasts across the four thin sections were considered for further investigation. Initially, this included high‐resolution BSE imaging of each clast (e.g., Figure 5) to aid with clast type identification. A range of material was identified including apparent igneous lunar Mg‐suite fragments, impact melt breccias, granulitic breccias, mare basalts, glass beads, feldspathic fragmental breccias, regolith breccias, granophyric material (likely from High Alkali Suite), and clasts with sulphide‐fayalite intergrowths.

The following inspection of the high‐resolution BSE images, 33 clasts were selected for EPMA analysis to precisely determine their mineral chemistry.

#### 3.4.1 Mg‐Rich Mafic Phases

**Figure 6:**
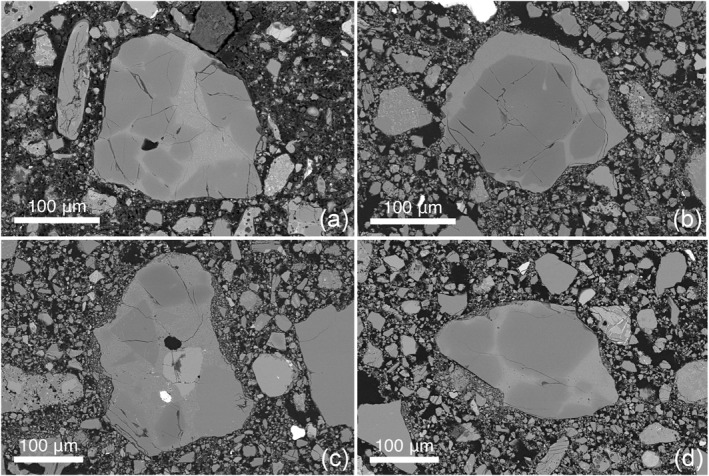
Clasts of Mg‐rich olivine hosted in feldspathic glass. (a) 73002,6012 ROI 058; (b) 73002,6013 ROI 052; (c) 73002,6013 ROI 017 which also contains a metal grain; (d) 73002,6014 ROI 005.

**Figure 7:**
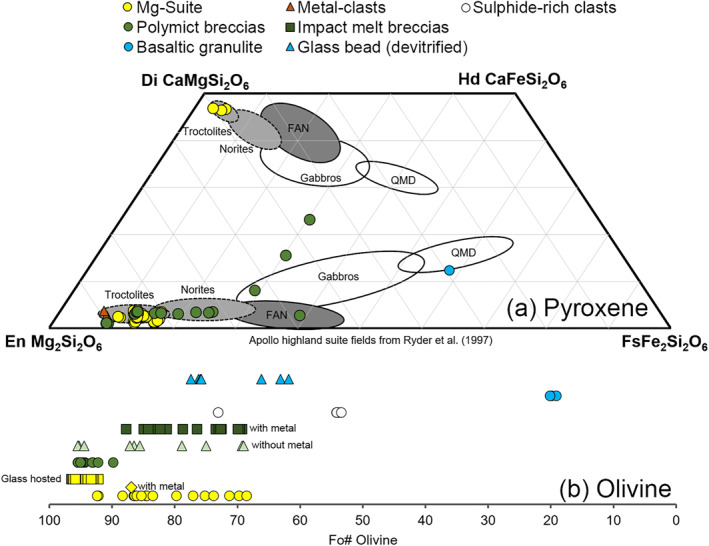
Mafic mineral chemistry of selected clasts in 73002 based EPMA analysis. (a) Pyroxene compositions compared with lunar highland rock fields (Ryder et al., 1997), where QMD = quartz monzodiorite, (b) range of olivine Fo compositions. Colors correspond to different rock types (e.g., yellow is Mg‐Suite) and shapes correspond to distinct groups identified within each rock type (e.g., yellow squares are glass hosted Mg‐suite clasts).

**Figure 8:**
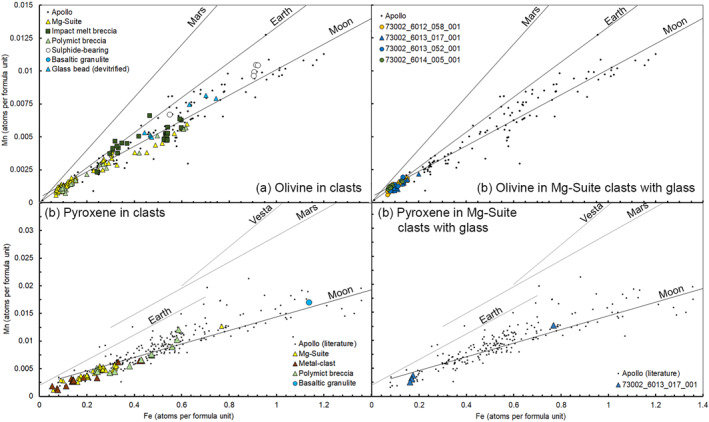
Mn versus Fe in atoms per formula unit in olivine, based on 4 atoms per formula unit (a and b) and pyroxene, based on 6 atoms per formula unit (c and d) using EPMA analysis of clasts and mineral fragments in 73002. Data are compared with Apollo minerals in different rock types (from database of Jolliff et al. (2006)) and planetary basalt Mn/Fe trends (Papike et al., 1998).

**Figure 9:**
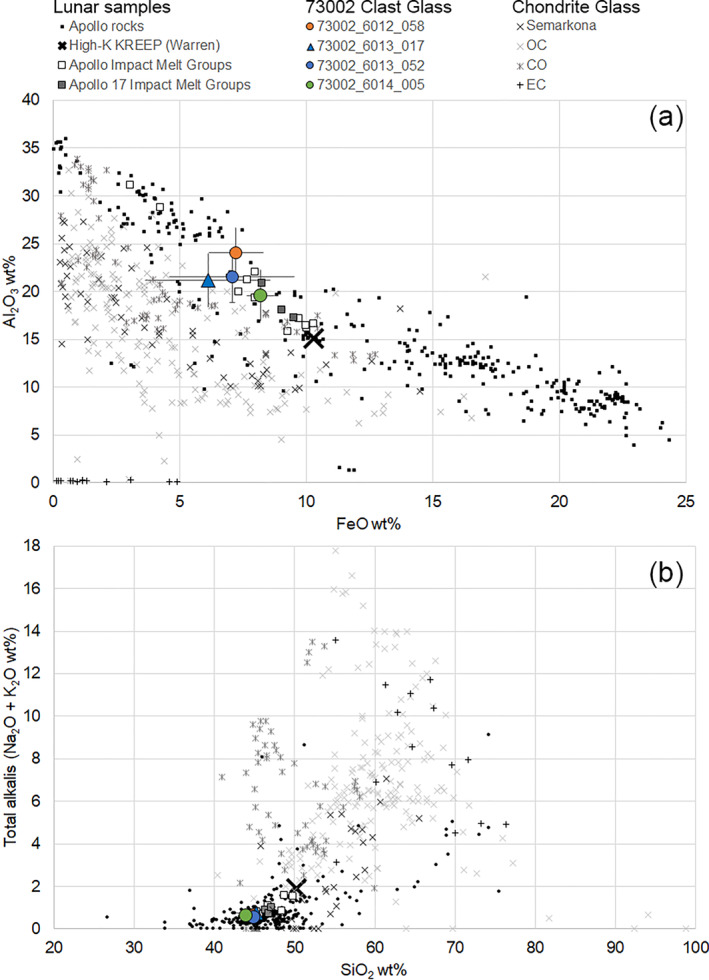
EPMA data showing the average chemistry of glass in the Mg‐rich clasts shown in Figure 6. The glass is compositionally distinct (lower alkalis and SiO_2_, higher Al_2_O_3_ and FeO/MnO) from chondrite meteorite chondrule glass (data from that compiled in Brearley and Jones (1998)), and is more similar to Apollo rocks and impact melts (data compiled in Jolliff (1998) and Jolliff et al. (2006)). Error bars represent 2 stdev.

Using EPMA data, we identified a distinct group of clasts that all contained Mg‐rich olivine (Fo_92.2‐96.5_) (Figures 6 and 7). The clasts have olivine Fe/Mn ratios similar to lunar materials (Figure 8b), and feldspathic glass compositions (average normative Mg#69–80, An#93–94, 0.06–0.10 wt% K_2_O, 0.47–0.66 Na_2_O wt%, FeO/MnO = 70–80) that are similar to lunar impact melts and dissimilar from chondrule mesostasis glass (Figure 9). Thus, the clasts are most likely lunar Mg‐suite material, and are not fragments of asteroidal meteorites. These olivine compositions are some of the most magnesian lunar olivine measured to date, with only an Fo_96_ olivine previously measured in a dunite clast from 76035 collected from Station 6 near the North Massif (Ryder et al., 1997). Other olivine clasts from Apollo 17 impact melt breccias have also been reported to have compositions of up to Fo_96_ (Shearer & Papike, 1995; Shearer et al., 2002). We interpret these clasts as fragments of Mg‐Suite dunite parent rocks that were excavated from depth and mixed into an impact melt sheet, which has been sampled by the South Massif deposit.

#### 3.4.2 Metal Fragments

**Figure 10:**
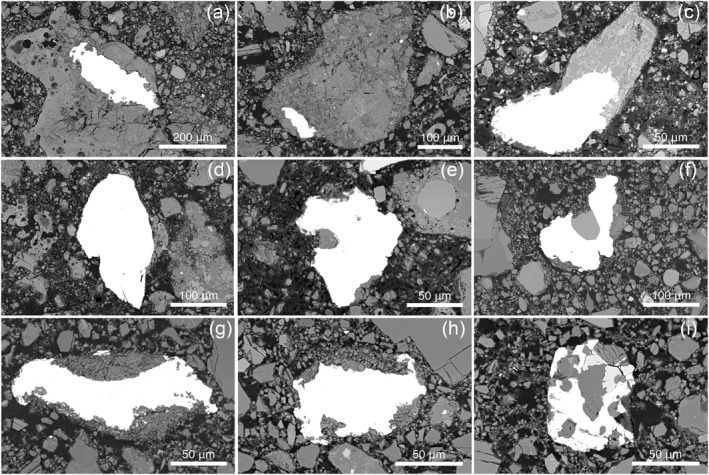
Fe,Ni metal fragments found in the 73002 matrix and within agglutinates, breccias, and impact melt breccias. (a) 73002,6011 ROI 009, (b) 73002,6011 ROI 059, (c) 73002,6012_SEM_ROI_013, (d) 73002,6012_SEM_ROI_060, (e) 73002,6012_SEM_ROI_063, (f) 73002,6013_SEM_ROI_013, (g) 73002,6013_SEM_ROI_036 (h) 73002,6014_SEM_ROI_014, (i) 73002,6014_SEM_ROI_021.

**Figure 11:**
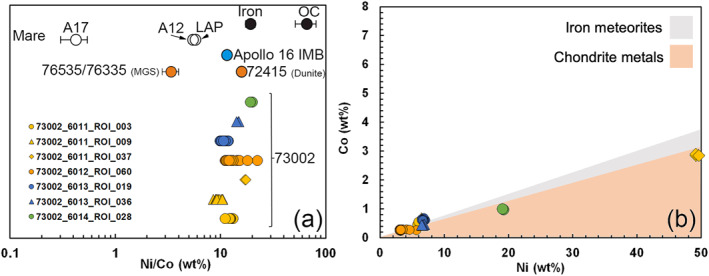
(a) EPMA data showing Ni/Co ratios of Fe,Ni metal fragments in 73002 compared with compositions of Fe,Ni metal in other lunar and meteoritic materials (data modified from Day (2020)), where LAP = LaPaz mare basaltic lunar meteorites, OC = ordinary chondrites, MGS = Mg‐Suite, Apollo 16 IMB = Apollo 16 impact melt breccias, Iron = iron meteorites. Samples are stacked in *y*‐axis to aid visual comparison, only. (b) Co versus Ni abundances in Fe,Ni metal fragments in 73002, illustrating their compositional variability with kamacite (low‐Ni) and high‐Ni compositions. Regions showing the compositions of chondrite metals (orange) and iron meteorites (gray) are after Day (2020).

As intact asteroid meteorite fragments on the Moon are rare, metal compositions in impact melts may represent impactor compositions mixed with target rock (see Joy et al., 2016). All of the silicate mineral fragments we found in association with metal grains (Figures 10 and 11) have lunar‐like compositions based on EMPA analysis. Measured Ni/Co ratios in Fe,Ni metal particles found in the 73002 matrix and within impact melt breccias and agglutinates (Figure 10) are similar to the composition of metal observed in Apollo 16 impact melt breccias (Figure 11a). Apollo 16 impact breccias have been interpreted to have formed from iron meteorite‐like impactor(s) (Fischer‐Gödde & Becker, 2012; Gleißner & Becker, 2017, 2020; Korotev, 1994; Liu et al., 2015; Worsham & Kleine, 2021). This might suggest a commonality between some impactor types that formed the impact melts sampled at both Apollo 16 and Apollo 17 (see also Gleißner & Becker, 2017).

### 3.5 Notable Clasts of Other Types of Lunar Lithologies

When inspecting clasts of interest to see if they were of meteoritic origin, we were also able to identify clasts of different lunar rock types that may be of use for other future scientific investigations. The QEMSCAN data collected can easily be used to identify the location of such clasts within the continuous core thin sections. Our interpretation of these clast types was based on both textural and chemical data and therefore correlated with common lunar clast types in the lunar literature.

**Figure 12:**
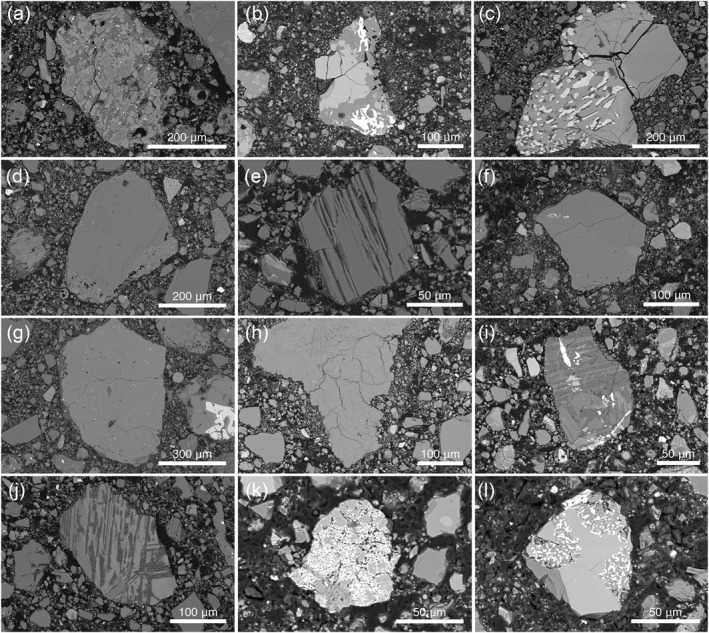
Clast types found in the 73002 core including mare basalts 73002,6011 ROI 004 (a), 73002,6012 ROI 023 (b), and mare basalts with symplectite textures 73002,6014 ROI 008 (c); mineral fragments likely sourced from Mg‐Suite parent rocks, such as mineral debris in impact melt clast 73002,6013 ROI 004 (d), brittle fractured olivine 73002,6013_SEM_ROI_041 (e), olivine‐pyroxene complex with fine grained symplectite spinel 73002,6012 ROI 042 (f), magnesian exsolved pyroxene 73002,6013_SEM_ROI_007 (g), norite clast (plagioclase and orthopyroxene) 73002,6012 ROI 003 (h); evolved clasts 73002,6012 ROI 015 (i) and 73002,601 ROI 043 (j); Sulphide‐rich clasts 73002,6012_SEM_ROI_035 (k) and 73002,6012_SEM_ROI_036 (l).

**Figure 13:**
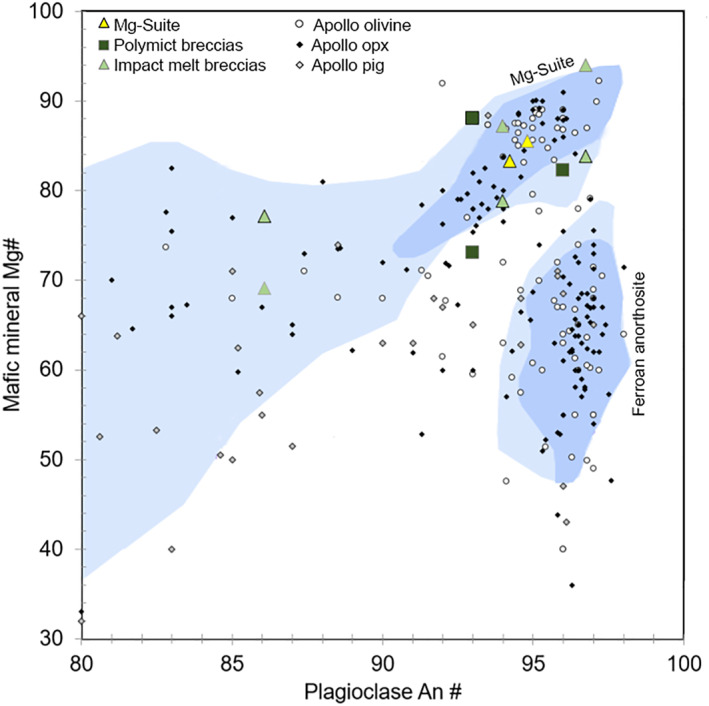
Mafic mineral (pyroxene = symbols with gray outlines, olivine = symbols with black outlines) Mg# compared with plagioclase An# in 73002 clasts. Data are compared with mineral‐pairs in pristine Apollo rocks (Warren, 1993), where the Mg‐Suite and FAN fields are from Yamaguchi et al. (2010). Shades of blue within the Mg‐Suite and Fan fields represent an arbitrary scale of “confidence in pristine character” after Warren (1993). The scale ranges from a confidence value of 9 for the most pristine samples, down to a value of 3 for the most unlikely pristine samples. Regions with “confidence” ⩾7 shown are shown in blue, and “confidence” ⩾6 are shown in light blue (see Yamaguchi et al., 2010 for more detail).

We found a range of polymineralic clasts of potential Mg‐suite material including dunites, norites, and gabbronorites (Figures 12 and 13). The Mg‐suite mineral debris showed a high degree of brittle fracturing compared with other mineral clasts (Figure 12). These highly fractured mineral clasts were observed in all four thin sections and ranged in size up to approximately 300 μm. Further high resolution BSE images of these clasts can be found in the accompanying data repository (https://doi.org/10.48420/c.7090330).

We also identified several clasts of more highly evolved rocks, such as felsite (Figure 12). These clasts showed a high proportion of K‐feldspar (or K‐rich glass) and quartz (or silica glass) with granophyric textures, or were found as fragments of albitic plagioclase within regolith breccias (Figure 12). Other high alkali suite (HAS) rocks collected at the Apollo 17 landing site tend to be gabbronorites, noritic granulites, and troctolites rather than granitic or quartz monzodiorites, although James and Hammarstrom (1977) describe a 20 mg felsite clast (#43,3) found in impact melt breccia 73215, also collected from the light mantle deposit at Station 3.

As sulphides were a mineral of interest when looking for clasts of meteoritic origin, we identified several clasts with interesting sulphide textures (Figure 12). Several of the textures look similar to the sulphide replacement textures reported by Shearer et al. (2012) in Apollo 16 highland rocks. Shearer et al. (2012) speculated that such textures could be related to interaction with a S‐rich vapor. It is unclear whether similar S‐rich vapor mobility processes occurred in the regolith at the Apollo 17 site, or if these rocks have the same origin with sulphide replacement being common in ferroan anorthosite (FAN) and Mg‐suite rocks excavated by the Imbrium impact and sampled at both landing sites.

**Figure 14:**
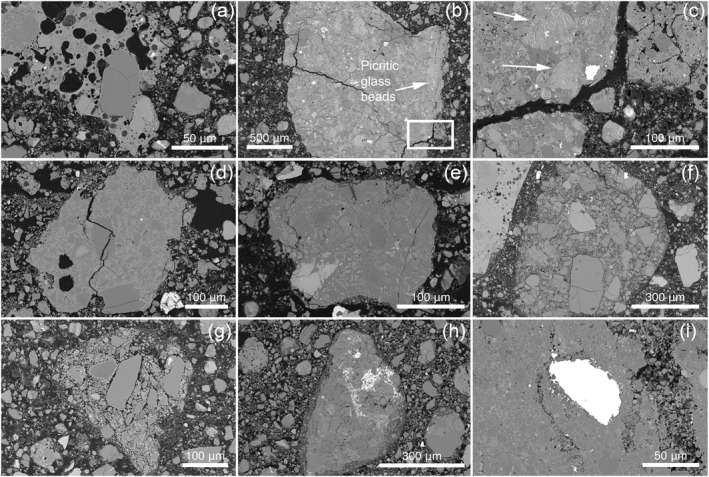
(a) Agglutinate 73002,6012 ROI 61; (b and c) regolith breccia clast 73002,6012 ROI 049 containing fragments of devitrified volcanic glass bead debris; (d) Clast‐bearing melt breccia 73002,6011_SEM_ROI_047; (e) crystalline impact melt clast 73002,6011_SEM_ROI_026; (f) fragmental polymict breccia 73002,6012_SEM_ROI_032; (g) fragmental polymict breccia 73002,6012_SEM_ROI_008; (h) fragmental polymict breccia 73002,6014_SEM_ROI_009; (i) Metal‐bearing feldspathic clast‐bearing impact melt clast 73002,6011_SEM_ROI_056, showing a close up of a portion of the large feldspathic melt clast discussed in Section 3.4.2.

Numerous varieties of impact melt and melt breccia textures were identified during the search for clasts of meteoritic origin. These included: impact melt clasts with high‐Mg olivine of potential Mg‐suite material; clast‐laden metal bearing impact melts; glass‐rich and metal‐rich impact melts; and metal‐bearing granulitic breccias (Figures 14d–14i). Likewise, clasts of regolithic origin such as agglutinates and regolith breccias (Figures 14a–14c) and mare basaltic material (Figure 12) are found distributed throughout the 73002 core.

## 4 Discussion

### 4.1 Variations in Modal Mineralogy With Depth in the Continuous 73002 Core

**Figure 15:**
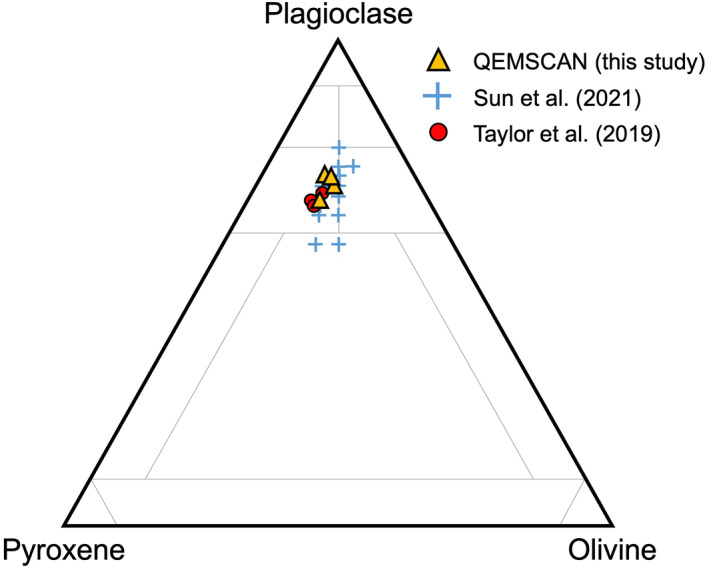
Ternary diagram plotting mineral modal abundances (after Sun et al. (2021)). This diagram is intended to represent mineral proportions and not lunar rock types although those regions are depicted for reference (Stöffler et al., 1980). Radiative transfer modeling using a hyperspectral profile through the core is represented by blue crosses (Sun et al., 2021). Apollo 17 Station 3 soils measured using XRD are represented by red symbols (Taylor et al., 2019).

The variability in modal mineralogy with depth across the 73002 core is gradual, with no observable rapid changes in composition or obvious stratigraphic boundaries (Figure 2). Normalized abundances of olivine, pyroxene, and plagioclase in the 73002 core thin sections are comparable to modal abundances calculated via spectral imaging of 73002 (Sun et al., 2021) and X‐Ray diffraction (XRD) analysis of other soil samples collected at the Apollo 17 Station 3 (Taylor et al., 2019) (Figure 15).

Olivine (∼5%–6%), pyroxene (7%–8%), and ilmenite (∼1%–2%) abundances are relatively consistent with depth across the four thin sections. Mafic minerals such as olivine, pyroxene, and ilmenite are the dominant carriers of FeO and TiO_2_; these oxides have been analyzed for the full length of 73002 in other studies using imaging and bulk chemical analysis techniques (inductively‐coupled‐plasma mass spectrometry), with FeO abundance varying from 7 to 9 wt.% and TiO_2_ varying from 1 to 2 wt.% (Neuman et al., 2023; Sun et al., 2021). The minimal variability in concentration of these elements suggests that the 73002 core sample did not capture much of a variation in sub‐surface stratigraphy of the regolith at Station 3 (Sun et al., 2021).

We observed variability in the proportion of other major lunar mineral phases, such as plagioclase, in our QEMSCAN data (Figure 2 and Table 1). The proportion of plagioclase increases with depth within the core from ∼29% in 73002,6011 (0–4.7 cm depth) to 39% in 73002,6014 (14.3–18.4 cm depth). Reflectance spectral signatures (which identifies feldspathic phases) of the 73002 core also show an increase of feldspathic material with depth (Morris et al., 2022). These findings indicate that the amount of feldspathic material increases with depth, and in turn, the proportion of mafic material (i.e., mare basalt clasts and mafic minerals) decreases with depth. Neuman et al. (2021, 2022) also reported a decrease in the calculated quantity of mare basalt components in the 73002 core with depth and an increase in anorthositic norite components (i.e., non‐basaltic and non‐impact‐melt breccia materials of the highlands massifs at Apollo 17).

The proportion of glass (a group which includes mafic glass, feldspathic glass, KREEP glass, and volcanic glass bead compositions) gradually decreases from 55% in 73002,6011 (0–4.7 cm depth) to 44% in 73002,6014 (14.3–18.4 cm depth). We also observed a sharp decrease in the proportion of agglutinates from 6% in 73002,6011 (0–4.7 cm depth) to 2% in 73002,6012 and <1% in 73002,6013 and 73002,6014 (Figure 3a). This correlates both with the higher space maturity estimates for the upper portion of the 73002 core from bulk sample I_s_/FeO measurements (Morris et al., 2022; Sun et al., 2021), and the proportion found in the <1 mm sieved fractions of bulk soil that show a higher proportion of agglutinates in the upper 5–6 cm of the core (Simon et al., 2024). A decrease in glass content with depth is consistent with investigations into space maturity of the core (Morris et al., 2022; Sun et al., 2021), which show that the upper 10–15 cm of the core (thin sections 73002,6011 to 73002,6013) is more mature and sub‐mature (i.e., glass‐rich) than the lower proportion of the core (thin section 73002,6014).

### 4.2 Variations in Clast Type and Size Distribution With Depth

The use of processors within the QEMSCAN software allowed for the separation of clasts within the continuous core thin sections and categorizers were used to group clasts into different clast types based on modal mineralogy/constituent phases. This allowed us to investigate how clast types and particle sizes varied with depth (Figures 3 and 4). This question has also been addressed in other studies of 73002 using electron microscopy to analyze individually mounted clasts from sieved portions of bulk <1 mm soils from different depths (Cato et al., 2022; Simon et al., 2022, 2024). Because our clast type definitions are limited to minerals/phases and cannot incorporate rock textural definitions, the groups in this study are not all comparable to those in Simon et al. (2024). However, there are certain clast type groups (e.g., monomineralic clasts, basaltic clasts) that are comparable, and from this we can see similarities between QEMSCAN‐derived abundances and those reported in Simon et al. (2024).

Simon et al. (2024) reported mare basalt clasts to be present at all levels within the bulk soil and we also identified polymineralic basaltic clasts in each of the four continuous core thin sections. The proportion of basaltic clasts in the QEMSCAN data is highest in 73002,6011 at 6% (Figure 3a, see some examples in Figure 12). In deeper samples the proportion decreases to <5%, reaching as low as 3.5%. This correlates with the reported decrease in mare basalt abundance with depth reported by Neuman et al. (2021, 2022).

Within the QEMSCAN data, we observed an increase in the proportion of plagioclase mineral fragments with depth from ∼9.5% to ∼17% (Figure 3). Simon et al. (2024) describe how the proportion of plagioclase fragments is higher than that of olivine and pyroxene, a feature that is also found in the QEMSCAN data (Figure 3). Both observations correlate with the increase in anorthositic norite components with depth proposed by Neuman et al. (2021, 2022, 2023) and the increase in reflectance spectral signature observed by Morris et al. (2022).

Overall, the variations in clast type abundance show non‐mare (i.e., all groups except basaltic clasts and basaltic glasses) clasts to be the dominant clast types (Figure 3). The higher proportion of non‐mare material is consistent with material derived from the south massif (Simon et al., 2024). As with the modal mineralogy results, the clast types are still relatively consistent with depth and do not show evidence of stratigraphic discontinuities (i.e., horizons marked by sharp changes in clast abundance) within the 73002 sample.

We also report particle size abundance with depth for all clasts within the core (Figure 4). As expected, based on the nature and texture of the core sample and the resolution of the QEMSCAN analysis (10 μm per pixel), the vast majority of clasts were <90 μm (Figure 4). As these clasts are only a few pixels in size in the QEMSCAN data set, the identified mineralogy of these clasts is also not at a high enough spatial resolution to be able to classify them into rock types based on mineralogy alone, so only clasts >150 μm were considered when looking at clast type distribution through the core. Overall, the particle size distributions per sample for clasts >150 μm in size show the same trends across each thin section, with the highest proportion of clasts being in the 150–250 μm category (>70%), with increasingly smaller proportions in the 250–500 μm (>30%) and 500–1,000 μm (>5%) categories. Once again, a similar trend is seen between all thin sections showing no variation with depth in the core.

Particles >1,000 μm made up only 0.008% of the total number of particles identified, which equates to 44 clasts in total in all the thin sections (Figure 4). Clasts >1,000 μm were not reported by Simon et al. (2024) as clasts larger than >1,000 μm were manually removed from the bulk soil during processing of the 73002 (Gross et al., 2023). Some clasts >1,000 μm still remain in the thin sections as these were prepared from an undissected portion of the core running its entire length. Of the clasts >1,000 μm, 18 are 10%–40% feldspathic glass clasts, 15 are 40%–80% feldspathic glass clasts, 6 are <10% feldspathic glass clasts, 3 are basaltic clasts, 1 is a mafic glass > feldspathic glass clast, and 1 is a plagioclase mineral fragment. There are no identifiable trends in clast types for clasts >1,000 μm with changing depth through the core.

### 4.3 QEMSCAN as a Method of Identifying Clasts of Extra‐Lunar Meteoritic Origin

The fine grained and complex nature of the 73002 continuous thin sections provided a challenge for the QEMSCAN systems, which were originally designed to deal with mining industry samples. Despite this, QEMSCAN provided all the data needed to identify potential clasts of meteoritic origin. The ability to produce maps of minerals of interest drastically reduced the time needed to inspect each sample. One of the limitations of routinely using QEMSCAN to identify clasts of meteoritic origin is the availability of the system and the time required to run samples, approximately 24 hr per thin section for the 73002 thin section, at 10 μm per pixel. QEMSCAN can run at a higher resolution than 10 μm per pixel, down to about 1 μm per pixel, however given the time required to run samples as large as the 73002 thin sections, this would not be feasible. If only mineralogical data is required, QEMSCAN can be run faster at a much coarser resolution of 50 μm per pixel, without any effect on calculated modal mineralogy (e.g., Neave et al., 2017).

The time required to perform analyses is lengthy, but a wide variety of useful data are produced. QEMSCAN provides mineral maps, element maps, BSE maps, and the ability to export the EDS spectra for each pixel within the map. Such a wealth of data allowed us to make observations beyond the goal of locating any clasts of meteoritic origin, without the need for any further analysis. In addition, the mineral phase maps produced can be used in future studies to help locate clasts of different lunar rock types. As such, we believe that QEMSCAN and other similar methods of non‐destructive automated mineralogy have the potential to provide a wealth of data for existing and future lunar samples.

Previous studies have shown that surviving fragments of meteoritic origin within Apollo and lunar meteorite samples are extremely rare (Day et al., 2006; Jolliff et al., 1993; Joy et al., 2012, 2020; Quaide et al., 1971; Rubin, 1997; Zolensky, 1997). Therefore, the lack of meteoritic fragments identified within the 73002 continuous thin sections in this study is not a surprise. Mostly likely, the impactors were completely vaporised leaving only the chemical signature of the impactors added to the regolith in the form of volatile elements and highly siderophile elements (see Joy et al., 2016 for overview).

Neuman et al. (2023) conducted elemental analysis using EPMA and inductively coupled plasma mass spectrometry (ICP‐MS) on powdered subsamples of the 73002 core at 0.5 cm intervals. With the measured elemental compositions, Neuman et al. (2023) used an error‐weighted, linear‐least‐squares approach to determine the proportions of different lithologic components (high‐Ti mare basalt, orange glass, noritic breccia, anorthositic norite, and a volatile‐free CM chondrite) within the 73002 regolith. Their results suggest that an average of 0.5% volatile‐free CM‐chondrite component is required to resolve the proportion of highly siderophile elements measured in the 73002 core samples (Neuman et al., 2023). While these results show that 73002 likely has some added meteoritic chemical component within it, our study suggests this is probably not in the form of intact meteoritic fragments.

However, there is still a possibility that fragments of meteoritic origin remain in the 73002 continuous thin sections. Although we automated as much of the search process as possible, our method required the manual process of determining which clasts were of interest. Where possible, we ensured that multiple authors of this study reviewed the selected clasts of interest to mitigate the chance of clasts of meteoritic origin not being selected for further BSE and EPMA analysis. The types of meteoritic clasts that were most susceptible to being overlooked were those with a high proportion of carbonaceous material or organics. As samples are carbon coated to facilitate QEMSCAN analysis, the QEMSCAN software does not report areas of high carbon as this is considered background. As such, the identification of these clast types was potentially less effective compared with other groups such as iron meteorites that would be easily detected. In addition, this set of sections represents only one thin slice of material from the 73002 core, and looking at additional thin sections from this core and the deeper 73001 core could offer additional opportunities to find any existing meteoritic clasts.

## 5 Conclusion

QEMSCAN is a powerful non‐destructive tool for collecting a range of mineralogical and textural data from the Apollo 17 73002 continuous core thin sections. We developed a semi‐automated method for searching for clasts of meteoritic origin using processors within the QEMSCAN software. We produced phase maps and element maps for each of the thin sections, which also included phase maps highlighting minerals of interest in the search for clasts of meteoric origin. We used these maps to identify over 200 clasts of interest that required further investigation. Detailed investigation of the clasts of interest revealed a wide range of lunar lithologies present, including; a distinct group of clasts that all contain Mg‐rich olivine (Fo_92.2‐96.5_), polymineralic clats of Mg‐suite material, highly evolved felsite clasts, and clasts with interesting sulphide textures. A review of high‐resolution BSE imaging of the clasts revealed that 33 clasts warranted further EPMA analysis to determine their origin. Ultimately, all clasts analyzed using EPMA showed element abundances consistent with a lunar origin. Other geochemical studies of the 73002 core suggest the presence of a meteoritic signature mixed within the regolith, but based on the samples analyzed in our study, the results indicate that this material is not present in the form of lithic grains.

We also extracted raw data from each pixel of the phase maps to determine the modal mineralogy with depth within the core. Our results did not reveal any stratigraphic layers within the 73002 core, although there were changes in plagioclase and glass abundances associated with soil maturity, and a decrease in mare basalt component with depth, an observation corroborated by other studies of 73002. We were able to separate individual clasts >150 μm from the phase maps, which allowed us to investigate the proportion of different clast types with depth. The proportion of agglutinates was higher in the shallowest thin section (73002,6011) than in the other three sections, also linking to the top 5–6 cm of the core being more mature.

Consistencies in the modal mineralogy and clast types with depth, and the lack of stratigraphy, potentially reflect mixing of material during the landslides off the South Massif above Station 3. The dominance of non‐mare material within all four thin sections is also consistent with the material being sourced from the South Massif. Further studies of the non‐mare clasts present within the core, potentially guided by QEMSCAN mineral maps, may provide further insight into the rock types present within the South Massif. Further work will use the methods developed here to also examine the 73001 continuous core thin sections. This method could also be applied to future returned lunar core and rock samples to both search for clasts of meteoric origin and collect a range of mineralogical and textural data.

## Supporting information

Supporting Information S1

## Data Availability

Additional details are reported in Supporting Information S1. The figures and numerical data used for in the study (sample images, QEMSCAN data, EPMA Data) are stored in a figshare online repository available in Bell et al. (2024).
