# Supplementary material for: Automated Mineralogy Analysis of the Apollo 17 73002 Continuous Core Thin Sections Using QEMSCAN Mapping Techniques
Source: J Geophys Res Planets. 2024 Nov 30;129(12):e2024JE008359. doi: 10.1029/2024JE008359 (PMC11608088; doi:10.1029/2024JE008359)
Supplement: Supplementary file 1 — Supporting Information S1 [file JGRE-129-0-s001.pdf]

JGR: Planets

Supporting Information for

Automated mineralogy analysis of the Apollo 17 73002 continuous core thin sections using QEMSCAN mapping techniques

S. K. Bell[1,2*], K. H. Joy[2], M. Nottingham[2,3], R. Tartèse[2], R. H. Jones[2], J. J. Kent[4] and C.K. Shearer[5,6] and the ANGSA science team[7]

[1]Stratum Reservoir AS, Nikkelveien 13 N-4313, Sandnes, Norway

[2]Department of Earth and Environmental Sciences, University of Manchester, Oxford Road, Manchester, M19 3PL, UK

[3]School of Geographical & Earth Sciences, University of Glasgow, Glasgow, G12 8QQ

[4]GeoControl Systems Inc., Jacobs JETS Contract, NASA/JSC, USA

[5]Department of Earth and Planetary Science, Institute of Meteoritics, University of New Mexico, Albuquerque, New Mexico 8713, USA

[6]Lunar and Planetary Institute, Houston TX 77058, USA

[7]ANGSA Science Team (list at https://www.lpi.usra.edu/ANGSA/teams/

Contents of this file

Text S1
Figures S1 to S3
Tables S1 to S3

Introduction

This document provides additional technical information about certain aspects of the QEMSCAN data processing for the analysis of the 73002 continuous core thin sections. For this study, we used an updated version of the SIP list used by Bell et al. (2020) that was specifically established for lunar samples, referred to as the 'Lunar SIP list'. Table S1 outlines some of the changes made to the original Lunar SIP list required due to the subdivision of the broad single lunar glass composition into different more detailed glass types (i.e., volcanic, or feldspathic or mafic impact derived glasses).

Clasts within the samples were separated using a combination of count rate thresholds and processors within the QEMSCAN software. Figure S1 provides an overview of the steps involved using count rate to identify the edges of clasts. Categorisers in the QEMSCAN software were used to split clasts into

different clast types based on composition. To do this, compositional definitions for the different clast types had to be entered. These included: monomineralic clasts (i.e. plagioclase, pyroxene, olivine and ilmenite), felsites, agglutinates, basaltic clasts, basaltic glasses and a number of categories classifying the breccia clasts based on the proportion of mafic and feldspathic glass present. Details of the definitions used can be found in Table S2.

Additionally, compositional plots of pyroxene (Fig. S2) and plagioclase (Fig. S3) using electron microprobe (EPMA) data are provided for each thin section. These were plotted using the same data available in tabular form in the accompanying data repository (DOI: https://doi.org/10.48420/c.7090330).

Included at the end of this document is a brief overview of the materials stored in the accompanying data repository (DOI: https://doi.org/10.48420/c.7090330). This includes full resolution, digital microscope images, back-scatter electron (BSE) images and maps, and QMESCAN mineral phase maps for all four thin sections.

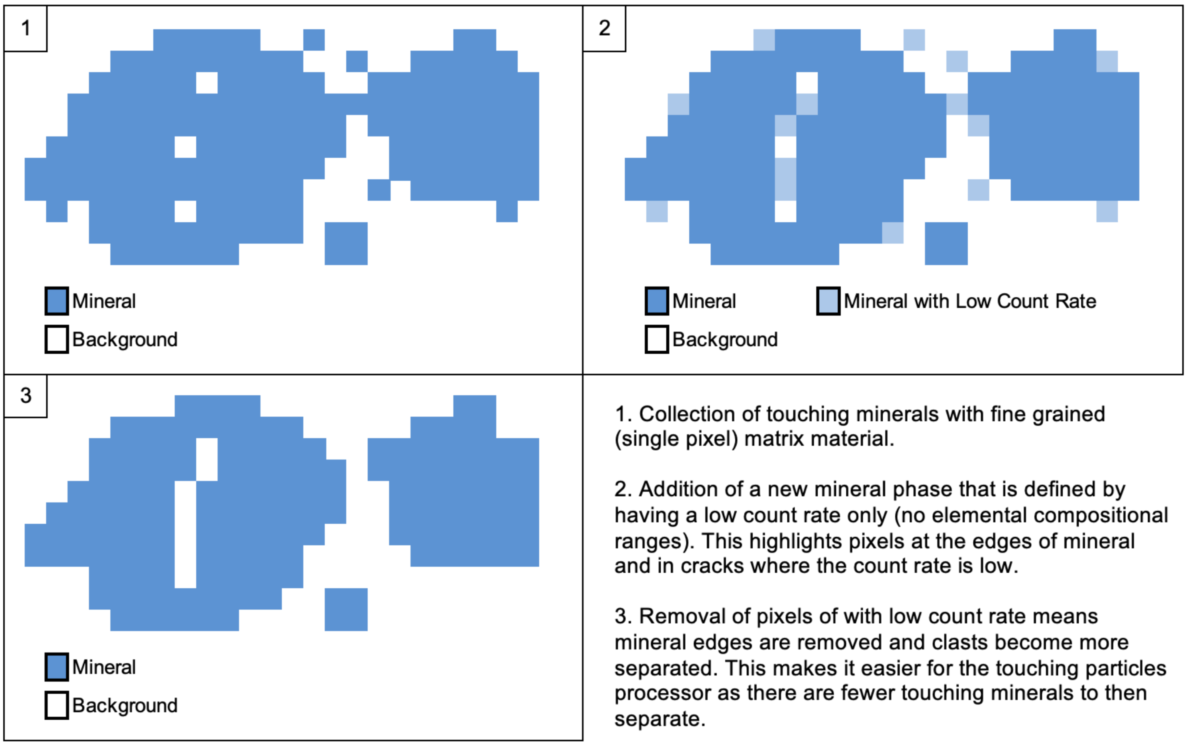

Figure S1. Schematic flow diagram detailing the steps involved with using count rate to identify the edges of clasts and help separate touching clasts.

	Very Low-Ti Glass		Low-Ti Glass		High-Ti Glass		Bytownite		Feldspathic Glass		Pigeonite		Mafic Glass	
	Min	Max	Min	Max	Min	Max	Min	Max	Min	Max	Min	Max	Min	Max
**O**	0	25	0	25	0	25	4.5	20	5	21.5	2.9	38.7	*0*	*30*
**Na**	*0*	*2.7*	*0*	*2.7*	*0*	*2.7*	*0*	*3.3*	-	-	-	-	-	-
**Mg**	5.3	15	2.3	15	1.9	15	*0*	*2.3*	*0*	*10*	*0*	*20*	*0*	*12.1*
**Al**	2	8	2	9.2	2	10.2	18.8	35	14.6	35	*0*	*4.4*	*0*	*17.7*
**Si**	24	36.1	19	36.1	17.7	36.1	23	41	21	41	23.4	43	*25*	*101.4*
**Ca**	4.96	19.1	4.96	21.1	2.7	20.5	15	32.1	14.1	30	4.9	22	*0*	*30*
**Fe**	15	34.2	15	34.2	14.4	33.6	*0*	*4.9*	2	20	5.1	42	*0*	*31.1*
**Ti**	*0*	*2*	2	10	9.8	20	*0*	*2.7*	-	-	*0*	*3.5*	*0*	*6.1*
**K**	*0*	*3.4*	*0*	*3.4*	*0*	*4*	*0*	*10.2*	-	-	*0*	*3.6*	*0*	*5*
**Cr**	*0*	*4.4*	*0*	*4.4*	*0*	*4.4*	*0*	*2.7*	-	-	*0*	*4.2*	-	-
**Mn**	*0*	*3.2*	*0*	*3.2*	*0*	*3.2*	*0*	*2.5*	-	-	*0*	*3.3*	-	-
**Ca/Al**	1	10	1	10	1	10	-	-	-	-	-	-	-	-
	May Contain: P(3), S(2.5), Cl(2.6), V(3.1), Co(4.9), Ni(3.3), Cu(4.3), Zn(4.4), As(5.8), Y(6), Zr(3.4), Nb(3.8), Ru(7.7), Rh(4.7), Pd(4.9)		May Contain: P(2.4), S(2.8), Cl(3), V(3.5), Co(4.9), Ni(3.3), Cu(4.3), Zn(4.4), As(5.8), Y(6), Zr(4), Nb(3.8), Ru(7.7), Rh(4.7), Pd(4.9), Ag(5.2)		May Contain: P(3.5), S(2.5), Cl(2.8), V(3.8), Co(4.9), Ni(3.3), Cu(4.3), Zn(4.4), As(7.4), Y(6), Zr(3.9), Nb(3.8), Ru(7.7), Rh(4.7), Pd(6.8), Ag(5.5)		May Contain: F(2.1), P(5.7), Cl(2.5), V(2.9), Co(3.1), Ni(4), Cu(3,8), Zn(4), As(4.9), Y(7), Zr(3.3), Nb(3.2), Ru(3.9), Rh(4.8), Pd(4.6)		May Contain: Y(7), Ag(6), Sn(7), Sb(12.1), Te(15), La(7), Ce(7), W(10.4), Re(10), Os(10), Ir(11), Pt(11), Au(10), Pb(9), Bi(10), Th(7), U(9)		May Contain: P(2.3), S(2.4), Cl(2.2), V(2.6), Co(4.3), Ni(3.2), Cu(5.5), Zn(5.3), As(6.9), Y(6), Zr(4.1), Nb(3.6), Ru(4.1), Rh(5.3), Pd(4.5), Ag(4.9)		May Contain: Co(5.7), As(5.4), Y(7.1), Ru(7.4), Sn(7.6), Sb(15), Te(8.9), Ba(10.6), La(6.9), Ce(5.6), W(6.2), Re(6.2), Os(8.7), Ir(8.8), Pt(10.1), Au(9.2), Pb(9.5), Bi(9.3)	

Table S1. Compositional ranges of elements in major minerals and glass compositions in updated version of Lunar SIP list (Bell et al., 2020). Element ranges are defined using a maximum and minimum concentration. The concentration is relative to a maximum of 125%, imposed by the QEMSCAN software to accommodate low counting statistics. Within the mineral definition, elements are either set as "Must haves" or "May contain". This is to distinguish between elements that are essential to that mineral definition (i.e. "Must haves") and additional elements (generally lower in concentration) that may sometimes occur within that mineral but are not essential (i.e. "May contain"). Ratios between two elements can also be incorporated into mineral definitions, e.g. Ca/Al ratio. Minimum and maximum values for common major elements are provided. In some cases these elements may not be essential to all mineral definitions and so these values are represented by italics if they are a "May contain" element. Additional "May contain" elements are shown below each mineral definition with values in brackets denoting the maximum concentration allowed. The "May contain" elements often represent noise within the spectra rather than a true occurrence of an element, but their inclusion is required within the mineral definition in order to provide a match.

	QEMSCAN categoriser definition
**Plagioclase**	AreaPercent(“Short List”.Feldspar)>90
**Olivine**	AreaPercent(“Short List”.Pyroxene)>85
**Pyroxene**	AreaPercent(“Short List”.Olivine)>90
**Ilmenite**	AreaPercent(“Short List”.Ilmenite)>60
**Basaltic Glasses**	AreaPercent(“Long List”.Lunar High-Ti Volc Glass)+ AreaPercent(“Long List”.Lunar Low Ti Volc Glass)+ AreaPercent(“Long List”.Lunar VLT Volc Glass) + AreaPercent(“Long List”.Mare Impact Glass)+ AreaPercent(“Long List”.Ilmenite)>80
**Felsite**	AreaPercent(“Long List”.Quartz)+ AreaPercent(“Long List”.Orthoclase)+ AreaPercent(“Long List”.Labradorite)+ AreaPercent(“Long List”.Andesine)>40
**Agglutinates**	AreaPercent(“Long List”.Epoxy)+ AreaPercent(“Long List”.Background)>20 and AreaPercent(“Long List”. Epoxy)>0
**Basaltic clasts**	AreaPercent(“Long List”.Augite)+ AreaPercent(“Long List”.Pigeonite)+ AreaPercent(“Short List”.Olivine)>20 and AreaPercent(“Short List”.Ilmenite)>0 and AreaPercent(“Long List”.Highland Impact Glass)<15 and AreaPercent(“Long List”.En60)+ AreaPercent(“Long List”.En70)+ AreaPercent(“Long List”.En80)+ AreaPercent(“Long List”.En90)+ AreaPercent(“Long List”.Enstatite)<5 and AreaPercent(“Short List”.Feldspar)<60 and AreaPercent(“Short List”.Olivine)<30
**mafic>feld glass**	AreaPercent(“Long List”.Glass)>10 and AreaPercent(“Long List”.Mare Impact Glass)>AreaPercent(Highland Impact Glass)
**<10 feldspathic glass**	AreaPercent(“Short List”.Feldspar)+ AreaPercent(“Short List”.Olivine)+ AreaPercent(“Short List”.Pyroxene)>30 and AreaPercent(“Short List”.Highland Impact Glass)<10
**10-40 feldspathic glass**	AreaPercent(“Short List”.Feldspar)+ AreaPercent(“Short List”.Olivine)+ AreaPercent(“Short List”.Pyroxene)>5 and AreaPercent(“Short List”.Highland Impact Glass)<40
**40-80 feldspathic glass**	AreaPercent(“Short List”.Feldspar)+ AreaPercent(“Short List”.Olivine)+ AreaPercent(“Short List”.Pyroxene)+AreaPercent(“Long List”.Mare Impact Glass)>3 and AreaPercent(“Short List”.Highland Impact Glass)<80
**>80 feldspathic glass**	AreaPercent(“Long List”.Highland Impact Glass)>80

Table S2. Compositional definitions input into the categorisers in the QEMSCAN software to separate clats into different clast types. The “AreaPercent” function refers to the area percentage of a particular mineral within an individual clast. The terms “Short List” and “Long List” refer to the secondary mineral lists; the Long List being the full list of mineral definitions and the Short List being all mineral definitions grouped into broader mineral groups. The phase names detailed here are the customised names used in the Lunar SIP list modified after Bell et al. (2020). Clasts are matched on a first order basis, and so the order given here matches the order within the categoriser. Definitions were established on an iterative basis whilst referring to images of the clasts falling within each definition to make sure the definitions are suitable.

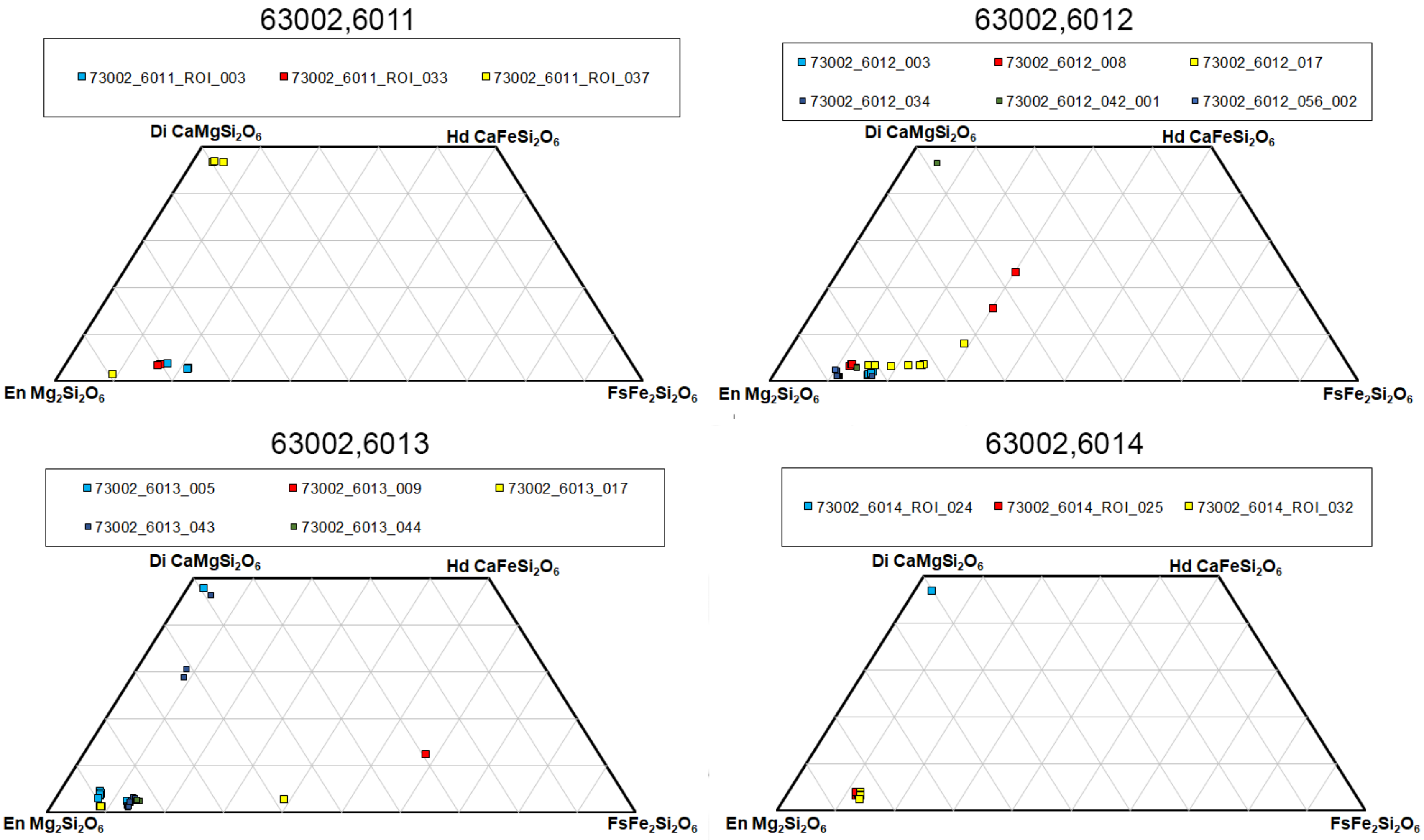

Figure S2. Pyroxene compositions measured in the four 63002 thin sections we have investigated. Colours used for symbol plotting do not denote a particular clast type.

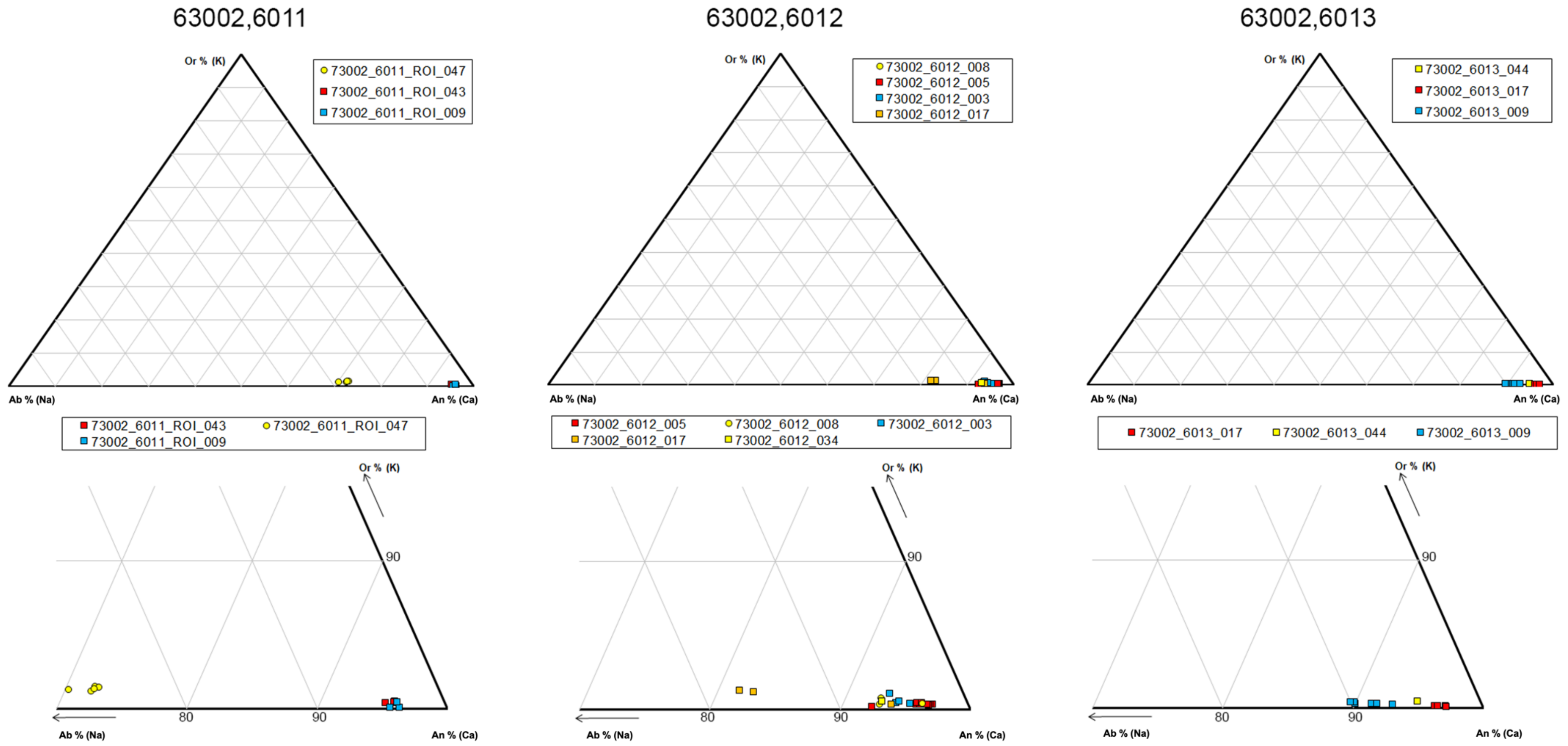

Figure S3. Plagioclase compositions measured in three out of the four 63002 thin sections we have investigated. We did not collect any plagioclase data in section 63002,6014. Colours used for symbol plotting do not denote a particular clast type. The top three plots show the complete plagioclase ternary with anorthite (Ca) - orthoclase (Or) - albite (Ab) end-members, and the lower three plots show just the An70-90 and Or0-10 range.

Text S1.

Figshare Data Repository Overview

Title: Apollo 17 ANGSA 73002 Core - QEMSCAN paper Datasets
Authors: S. K. Bell, K. H. Joy, M. Nottingham, R. Tartèse, R. H. Jones, J. J. Kent and C.K. Shearer and the ANGSA science team
DOI: https://doi.org/10.48420/c.7090330

This dataset provides additional supplementary material for this manuscript, including full resolution maps and images.

Contents:

Electron microprobe data for samples 73002,6011 to 73002,6014

QEMSCAN phase maps of samples 73002,6011 to 73002,6014

- _LongList - a primary lunar mineral list from which all secondary mineral lists are created
- _ShortList - a secondary mineral list that is a simplified version of the long list, grouping minerals into broader mineral groups (i.e. pigeonite and augite into pyroxene)
- _Datable - a secondary mineral list that highlights dateable minerals such as apatite and zircon
- _Feldspar - a secondary mineral list highlights different feldspar compositions
- _Felsite - a secondary mineral list highlights minerals common in lunar felsite clasts
- _Glass - a secondary mineral list a secondary mineral list highlights different glass compositions
- _MetalSulph - a secondary mineral list highlights metals and sulphides
- _Meteorites - a secondary mineral list highlights minerals commonly found in meteorites
- _Olivine - a secondary mineral list highlights different olivine compositions

Digital microscope images of samples 73002,6011 to 73002,6014

Back-scatter electron maps of samples 73002,6011 to 73002,6014

Back-scatter electron images of phases and clasts in samples 73002,6011 to 73002,6014

Name	Affiliation
Jessica Barnes	University of Arizona
Carolyn Crow	University of Colorado Boulder
Maryjo Brounce	University of California Riverside
Jeremy Boyce	NASA Johnson Space Center
Jed Mosenfelder	University of Minnesota
Tom Zega	University of Arizona
Zoë Wilbur	University of Arizona
Sean Pomeroy	University of Colorado Boulder
Iunn Jenn Ong	University of Arizona
Timothy Hahn	Jacobs NASA Johnson Space Center
Timmons Erickson	Jacobs NASA Johnson Space Center
Jamie Elsila Cook	NASA Goddard Space Flight Center
Daniel Glavin	NASA Goddard Space Flight Center
Jason Dworkin	NASA Goddard Space Flight Center
Jose Aponte	NASA Goddard Space Flight Center
Danielle Simkus	NASA Goddard Space Flight Center
Darby Dyar	Mount Holyoke College/PSI
Antonio Lanzirotti	University of Chicago
Stephen Sutton	University of Chicago
Molly McCanta	University of Tennessee, Knoxville
Elizabeth Sklute	Planetary Science Institute
Kees Welten	University of California Berkeley
Kunihiko Nishizumi	University of California Berkeley
Marc Caffee	Purdue University
Natalie Curran	NASA Goddard Space Flight Center
Barbara Cohen	NASA Goddard Space Flight Center
Sarah Valencia	University of Maryland
Catherine Corrigan	National Museum of Natural History
Emma Bullock	Carnegie Earth and Planets Laboratory
Kate Burgess	Naval Research Laboratory
Rhonda Stroud	Naval Research Laboratory
Brittany Cymes	Naval Research Laboratory
Alexander Sehlke	NASA AMES Research Center
Derek Sears	NASA AMES Research Center
Jeffrey Gillis-Davis	Washington University St. Louis
Richard Walroth	SLAC National Accelerator Laboratory
Thomas Kroll	SLAC National Accelerator Laboratory
Dimosthenis Sokaras	SLAC National Accelerator Laboratory
Hope Ishii	University of Hawaii
John Bradley	University of Hawaii
Roberto Colina-Ruiz	SLAC National Accelerator Laboratory
Charles Shearer	University of New Mexico
Bradley Jolliff	Washington University St. Louis
Mahesh Anand	Open University
James Carpenter	European Space Agency

Lars Borg	Lawrence Livermore National Laboratory
Katherine Joy	University of Manchester
Lindsay Keller	NASA Johnson Space Center
Paul Lucey	University of Hawaii
Clive Neal	University of Notre Dame
Noah Petro	NASA Goddard Space Flight Center
Harrison Schmitt	Harrison Schmitt Consulting
Richard Walker	University of Maryland
Alex Bradley	Washington University St. Louis
Adrian Brearley	University of New Mexico
William Cassata	Berkeley Geochronology Center
Roy Cristofferson	Jacobs NASA Johnson Space Center
Simon Clemett	Jacobs NASA Johnson Space Center
Aidan Cowley	European Space Agency
Catherine Dukes	University of Virginia
Kate Freeman	Penn State University
Amy Gaffney	Lawrence Livermore National Laboratory
Rhian Jones	University of Manchester
Randy Korotev	Washington University St. Louis
Thomas Kruijer	Lawrence Livermore National Laboratory
Gordon Love	University of California Riverside
Dayl Martin	European Space Agency
Matthias Maurer	European Space Agency
Alex Meshik	Washington University St. Louis
Alexandre Meurisse	European Space Agency
Richard Morris	NASA Johnson Space Center
James Papike	University of New Mexico
Rita Parai	Washington University St. Louis
Olga Pravdivtseva	Washington University St. Louis
Zachary Sharp	University of New Mexico
Justin Simon	NASA Johnson Space Center
Steven Simon	University of New Mexico
Corliss Kin Sio	Lawrence Livermore National Laboratory
Lingzhi Sun	University of Hawaii
Romain Tartèse	University of Manchester
Kathie Thomas-Keprta	Jacobs NASA Johnson Space Center
Michelle Thompson	Purdue University
Kun Wang	Washington University St. Louis
Josh Wimpenny	Lawrence Livermore National Laboratory
Michael Cato	University of New Mexico
Anthony Gargano	University of New Mexico
Mason Neuman	Washington University St. Louis
James McFadden	Purdue University
Dan Moriarty	NASA Goddard Space Flight Center
Giulia Magnarini	University College, London
Patrizia Will	Washington University St. Louis
Zhen Tian	Washington University St. Louis

Chris Yen	Washington University St. Louis
Abbey Flom	University of Hawaii
Chiara Ferrari-Wong	University of Hawaii
Jessika Valenciano	University of Notre Dame
Kamil Stelmach	University of Virginia
Grace Minesinger	University of Virginia
Grant Killian	University of Virginia
Paul Carpenter	Washington University St. Louis
Julian Rodriguez	Washington University St. Louis
Thomas Mitchell	University College, London
Peter Grindrod	Natural History Museum London
Karen Ziegler	University of New Mexico
James Dottin	Brown University
Stu Webb	University of Notre Dame
Samantha Bell	University of Manchester
Erick Cano	University of New Mexico
Linda Ziamanesh	University of Virginia
Jessica Oraegbu	University of Virginia
Adam Woodson	University of Virginia
Jillian Maxson	University of Virginia
Angelina Minocha	Washington University St. Louis
Ryan Ogliore	Washington University St. Louis
Caitlin Ahrens	NASA Goddard Space Flight Center
Francesca McDonald	European Space Agency
Advenit Makaya	European Space Agency
Nathan Bamsey	European Space Agency
Thomas Rohr	European Space Agency
Gianluca Casarosa	European Space Agency
Matteo Appolloni	European Space Agency
Robert Linder	European Space Agency
Yuriy Butenko	European Space Agency
Timon Schild	European Space Agency
Eoin Tuohy	European Space Agency
Cyrille Crespi	European Space Agency
Paul deMediros	European Space Agency
Benoit Andre	European Space Agency
Riccardo Biella	European Space Agency
Fiona Thiessen	European Space Agency
Ryan Zeigler	NASA Johnson Space Center
Juliane Gross	NASA Johnson Space Center
Charis Krysher	Jacobs NASA Johnson Space Center
Andrea Mosie	Jacobs NASA Johnson Space Center
Judith Allton	NASA Johnson Space Center
Scott Eckley	Jacobs NASA Johnson Space Center
Jeremy Kent	GeoControl NASA Johnson Space Center
Julie Mitchell	NASA Johnson Space Center
Cecilia Amick	Jacobs NASA Johnson Space Center

Ernest Lewis	Jacobs NASA Johnson Space Center
Romy Hanna	University of Texas, Austin
Richard Ketcham	University of Texas, Austin
David Edey	University of Texas, Austin
Evan O'Neal	Jacobs NASA Johnson Space Center
Francis McCubbin	NASA Johnson Space Center
Tabb Prissel	NASA Johnson Space Center
Tin Ho	University of Virginia
Kelsey Prissel	Jacobs NASA Johnson Space Center
Lauren Galien	University of Notre Dame
Hannah McLain	NASA Goddard Space Flight Center

Table S3. Members of the ANGSA Science team (list at https://www.lpi.usra.edu/ANGSA/teams/)
